# Necroptosis and Viral Myocarditis: Tumor Necrosis Factor *α* as a Novel Biomarker for the Diagnosis of Viral Myocarditis

**DOI:** 10.3389/fcell.2022.826904

**Published:** 2022-05-04

**Authors:** Jin Zhou, Jing Xu, Peng Li, Shan Sun, Yimiti Kadier, Shiying Zhou, Aijuan Cheng

**Affiliations:** ^1^ Tianjin Chest Hospital, Tianjin, China; ^2^ Hotan District People’s Hospital, Tianjin, China

**Keywords:** necroptosis, viral myocarditis, molecule mechanism, tumor necrosis factor α, diagnosis

## Abstract

Programmed cell death (PCD), including necroptosis, has emerged as a significant pathway in cardiovascular diseases. The infection of viral myocarditis (VMC) could cause cardiomyocytes degeneration, necrosis, and immune-inflammatory myocardial response. In this review, we summarized and evaluated the available evidence on the pathogenesis, molecule mechanism, diagnosis, and potential treatment strategies of viral myocarditis, with a special focus on the novel mechanism of necroptosis for cardiomyocytes death. Studies have shown that tumor necrosis factor-alpha (TNF-α) is an important cytokine involved in the activation of necroptosis; an elevated level of TNF-α is continually reported in patients suffering from VMC, implicating its involvement in the pathogenesis of VMC. It is of great interest to explore the clinical implication of TNF-α. We subsequently conducted a meta-analysis on the efficacy of serum TNF-α expression level and its diagnostic accuracy on acute viral myocarditis detection. Taken together, the review demonstrates a compelling role of necroptosis involved in the pathogenesis of VMC. Further, applying TNF-α as a serological marker for the diagnosis of VMC may be a useful strategy.

## 1 Introduction

Functional activities of all multi-cellular organisms, maintenance of a homeostatic balance between new cells and those damaged or unrequired cells, or to speak simply, regulating the number of cells, demand a constant effort of cell death (Galluzzi et al., 2009). Cell death is a common and important feature in the growth and development of tissues and organs. Normally, cells are removed in a controlled (programmed) manner, involving a series of biochemical and molecular events, leading to a turnover of damaged or unrequired cells that are supposed to be removed from the body (D’Arcy, 2019). Perhaps the most widely recognized forms of cell death have been described in terms of two types: apoptosis, known as programmed cell death, or necrosis, known as uncontrolled cell death (Kroemer et al., 2009). Necrosis is classically characterized as via an unprogrammed or accidental form that is caused by physical or chemical injury resulting in the rupture of the cell and cellular content leakage into the extracellular space, which ultimately causes the release of damage-associated molecular patterns (DAMPs) (Vandenabeele et al., 2010a). Instead, apoptosis, from a Greek term denoting leaves falling from a tree, is the most well-studied form of programmed cell death and has been characterized as a process controlled by multi-genes and somehow is referred to as programmed cell death (PCD) at the very beginning. Though it was previously thought that necrosis is passive and unprogrammed, research has now uncovered the caspase-independent processes that resemble necrosis. A new form of programmed cell necrosis, termed necroptosis, and alongside pyroptosis, ferroptosis, and cellular senescence, which is clearly distinct from apoptosis, has now been identified as one of the components of programmed cell death.

## 2 Overview of Necroptosis

Understanding the molecular mechanism of any form of PCD and its roles in disease pathogenesis and development is the key point of current research, which may contribute to effective biomarkers for diagnosis, new therapeutic targets, treatment strategies, and management options.

The word necroptosis is renamed to distinguish from necrosis that can be seen as accidental (unprogrammed) cell death that occurs in response to overwhelming chemical or physical insult, extreme physical temperature, pressure, chemical stress, or osmotic shock (Degterev et al., 2005). Necroptosis is the process of cellular self-destruction that is activated when apoptosis is otherwise prevented. However, the term “necroptosis” had not been generally adopted in the early period that “necrosis” or “necrotic cell death” was also used in the literature (Vandenabeele et al., 2010b). Morphologically, the programmed necrotic cells present a similar character of “necrosis” by cytoplasmic and organelle swelling, disruption of osmotic balance due to the extensive intracellular vacuolation, rupture of the plasma membrane, and cell death. Also, this release of cellular “adjuvants” from the necrotic cells promotes inflammation (Kono and Rock, 2008).

In 1997, Vercammen et al. found that triggering of the TNF receptors leads to necrosis of L929 cells while the Fas antigen results in apoptotic cell death, implicating that cell necrosis may also be regulated by a programmed form, and either necroptosis or apoptosis can occur in the same cell type within the pathways sharing the same cellular components (Vercammen et al., 1997). In addition, it is found that the pan-caspase inhibitor of zVAD-fmk (zVAD), a general inhibitor to block apoptosis, can induce necroptosis in L929 cells and the cell death is dependent on autocrine production of tumor necrosis factor-α (TNF-α) by Wu et al. (Wu et al., 2011). TNF-α has now been demonstrated to trigger programmed necrosis in several cell types (Holler et al., 2000; Lin et al., 2004; He et al., 2009). It was not very clear yet until 2009 that the only molecular component of programmed necrosis was identified as the protein serine/threonine kinase Receptor Interacting Protein 1 (RIP1) (Kono and Rock, 2008; Galluzzi et al., 2009). The role of kinases RIP1/RIP3 was identified in TNF-induced necroptosis in subsequent research (He et al., 2009; Vandenabeele et al., 2010b; Cho et al., 2011). Other cellular receptors that trigger the activation of necroptosis are death receptors of Fas/FasL and Toll-like receptors (TLR4 and TLR3) and cytosolic nucleic acid sensors such as RIG-I and STING, which can induce TNF-α and type I interferon (IFN-I) production promoting necroptosis in an autocrine feedback loop (Chen et al., 2018; Bertheloot et al., 2021).

Broadly speaking, necroptosis is confirmed as a new form of programmed cell death, distinct from necrosis that necrosis is not an uncontrollable passive process at all, but there is a possibility for a controllable form of cell death involving in PCD. Apoptosis was long thought to be the only regulated cell death pathway and the subject of intensive research for decades. On the contrary, its counterpart, necroptosis, has been investigated until recently that it can also be induced by TNF-α following its activation on tumor necrosis factor receptor 1 (TNFR1) (Table 1) (Pennica et al., 1984; Gon et al., 1996; Wang et al., 2008). Whereas activation of caspases represents the primary route of apoptosis, necroptosis represents an alternative form of PCD that is caspase-independent (Vandenabeele et al., 2010a). Apoptosis participates in limited individual or small clusters of cells when caspase-independent PCD contributes to the vital process of an immune response, osteogenesis, ovulation, and cellular turnover (Festjens et al., 2006; Sosna et al., 2014). Furthermore, the involvement of necroptosis has been widely implicated in the pathology of various diseases across the body including neurologic, renal, pulmonary, and gastrointestinal systems like pancreatitis (He et al., 2009; Zhang et al., 2009) and cancer; inflammatory diseases such as psoriasis, ulcerative colitis, and Crohn’s disease; autoimmune diseases; TIDM; as well as cardiovascular diseases like coronary atherosclerosis, heart failure, myocardial ischemia/reperfusion injury, and myocarditis (Karunakaran et al., 2016; Meng et al., 2016; Moe and Marín-García, 2016; Zhang et al., 2016; Zhou et al., 2018; Sun et al., 2019). Its role has also been identified in the investigation of cell damage by pathogens like vaccinia virus, Shigella and Salmonella, HIV. The programmed processes are important as they have been implicated in the pathology of many diseases (Liu et al., 2015; Yang et al., 2018). The concept of cell death and programmed cell death has dramatically changed over the years.

**TABLE 1:** Difference between necrosis, apoptosis, and necroptosis

	Necrosis	Apoptosis	Necroptosis
Cell death type	Non-programmed cell death	Programmed cell death	
Induced by	Physical/environmental factors outside the cell	Activation of instructions within cell DNA	Outside trauma or deprivation
Activated through	Passive and uncontrollable	Intrinsic pathway: oligomerization of the B-cell lymphoma-2 (BCL-2) family proteins BAK and BAX	Following the activation of the tumor necrosis receptor (TNFR1) by TNF-α; other receptors such as death receptors (i.e., Fas/FasL), Toll-like receptors (TLR4 and TLR3), and cytosolic nucleic acid sensors such as RIG-I and STING, which induce type I interferon (IFN-I)
		Extrinsic pathway: engagement of membrane receptors such as TNFR1, or Toll Like Receptors (TLRs)	
Caspase-dependent manner	Uncontrolled and passive process	Caspase-dependent	Caspase-independent
Occurred Site	Occurred in large fields of cells	Limited to individual or small clusters of cells	Occurred in vital process
Morphological Features	Loss of membrane integrity; cytoplasmic vacuole formation; ruptured mitochondria and lysosomal organelles; release of cytosolic content into extracellular regions	Maintainance of membrane integrity; apoptosis body was phagocytosed by macrophages or adjacent cells	Loss of membrane integrity; myocardial swelling; unclear and strong acidophil staining; pyknosis, karyorrhexis; karyolysis; inflammatory cell infiltration
Inflammatory response	Yes	No	Yes
Gene regulation	No	Yes	Yes
Protective role	—	Preventing cancer and regulating cell growth	DAMPs caused by necroptosis will alert surrounding cells of danger and promote inflammation
Promote disease progression	—	Excessive cell apoptosis can lead to serious diseases such as Parkinson’s disease and Alzheimer’s disease	Excessive cell necroptosis may contribute to inflammatory diseases such as psoriasis, ulcerative colitis, and Crohn’s disease

## 3 Epidemiology and Pathogenesis of Viral Myocarditis

The understanding of specific viruses affected myocarditis is still unfolding and its pathogenesis remains further investigation. Myocarditis is one of the clinically important cardiovascular diseases induced predominantly by viruses and also by other infectious agents including bacteria, protozoa, and fungi. The disorder is associated with poor prognosis when complicated by left ventricular dysfunction, heart failure, or arrhythmia (Ammirati et al., 2018). Because its specific mechanism has not been fully understood, the absolute incidence and prevalence are hardly recorded and the problem of misdiagnosis is always common. A retrospective study of 1.35 million deaths with certified causes reported myocarditis as the underlying cause of death in 639 cases (Kytö et al., 2007). Among patients visiting the emergency department between 1990 and 2013, the incidence of myocarditis was estimated to be 10–22 out of 100,000 individuals (Collaborators GBODS, 2015).

There are five common viruses associated with viral effect myocarditis that can be categorized as follows: the primary viruses are adenoviruses and enteroviruses such as coxsackie B or echoviruses; viscerotropic viruses, for example, parvovirus B19 have lifelong persistence (Dominguez et al., 2016; Van Linthout et al., 2018), though its exact mechanism may need to be investigated in that these viscerotropic viruses do not play a significant role to the etiology of adult myocarditis (Koepsell et al., 2012); human herpesvirus 6 (HHV6), Epstein–Barr virus and human cytomegalovirus of the lymphotropic viruses; viruses that can indirectly trigger myocarditis by the activation of the immune system, such as human immunodeficiency virus (HIV), influenza A virus, and influenza B virus; and family coronaviruses including the MERS-CoV, SARS-CoV, and SARS-CoV-2 that is recently found to also lead to myocarditis (Topol, 2020; Çınar et al., 2020). The incidence of viral myocarditis in any viral infection is estimated at 3%–6% (Narovlyanskaya and Winokur, 2020). The clinical importance of virus persistence has been demonstrated by Why et al. with higher in-hospital mortality at long-term follow-up (Why et al., 1994).

Research indicates that infection on the myocardium by enteroviruses occurs in three phases (Pollack et al., 2015). In Phase 1, the virus enters into myocytes and activates innate immune response within 7 days: the Coxsackie B viruses exhibit tropism for cardiomyocytes via the coxsackievirus and adenovirus receptor (CAR); the persistent viral replication leads to the dissolution of cardiomyocytes and to necrosis; immune responses can be subsequently activated. High levels of cytokines, such as tumor necrosis factor (TNF), interleukins, and interferons, are produced during this phase. Phase 2 may last for weeks (1–4 weeks), and during this phase, cytokine and chemokine are released; T cell-mediated clearance of virus-infected cells contribute to myocardial injury, necrosis, and production of interleukins and interferons. Commonly, there are two outcomes as viral titers decrease in phase 3. Some individuals experience complete resolution of myocardial injury and normalization of LV systolic function. However, in other patients, ineffective viral clearance and viral persistence throughout the entirety of the stage contribute to dilated cardiomyopathy, featured as chronic inflammation, cardiomyocytes degeneration, hypertrophy, interstitial fibrosis and global remodeling (Pollack et al., 2015).

## 4 Necroptosis and Viral Myocarditis

### 4.1 Link Between TNF-α and Viral Myocarditis

Cardiomyocyte is a kind of terminal differentiation cell with limited regenerating ability, and damage factors can induce cardiomyocyte death leading to cardiomyopathy and heart failure. Overwhelming cardiac inflammation and the loss of myocardium is an important pathogenic mechanism in the progress of myocarditis yet the induction of this mechanism is unclear (Kang and Izumo, 2003; Chen et al., 2014; Kühl and Schultheiss, 2014). It was recently found that TNF-induced PCD is considered deeply involved in the pathology and development of VMC that necroptosis may be a novel mechanism for cardiomyocyte death in the virus-induced acute myocarditis (Zhou et al., 2018).

Established models and clinical data have demonstrated a causal role for TNF-α in virus-induced myocarditis. (Wang et al., 2016; Zhou et al., 2018; Chen and Deng, 2019). In 2004, the TNF-α mRNA and TNF-α protein had been firstly reported to be significantly more present in viral myocarditis patients than in nonviral myocarditis, accompanying the histologic change of prominent myocardial necrosis compared to TNF-α-negative cases (Calabrese et al., 2004), suggesting that TNF-α mRNA and protein are overexpressed with viral myocarditis of any etiology. The cardiac TNF-α abundance during viral infection was observed (Glück et al., 2000; Wada et al., 2001; Gagliardi et al., 2004); TNF-α gene expression was seen in 80% of the viral positive myocarditis cases; immunohistochemistry for TNFR1/2 was overexpressed in the TNF-α-positive cases while the controls were always negative. Subsequent EMBs showed similar results that the same expression of the pathway was seen in the EMS (Calabrese et al., 2004). Immunostaining reported the marked positivity in the cytoplasm of myocytes. Histologically, the intensity of the inflammatory infiltrate was only significantly noted in the TNF-α-positive case, while there was no difference in terms of extension of fibrosis between all cases. In addition, clinical works demonstrated an association between depressed myocardial function and the elevated TNF-α mRNA and protein levels (Habib et al., 1996; Torre-Amione et al., 1996), suggesting that TNF-α plays an important role in the viral myocarditis pathology of the infiltration of inflammatory cells and activities of inflammatory mediators (Navarro and Mora, 2006). The exact mechanism of how TNF-α contributes to VMC is not well known, and previously it is suspected to be linked to immunity. Clinical studies demonstrated that the TNF-α serum in the VMC group was significantly higher than the controls (Wen et al., 2010; Wang et al., 2016; Chen and Deng, 2019). The possible mechanism currently demonstrated that a cytokine storm after the hyperactivation of the immune system in response to virus infection may cause multiple organ failure through inflammatory cell death; and depending upon the stimulus encounters, different forms of cell death are induced. (Malireddi et al., 2019; Christgen et al., 2020; Malireddi et al., 2020; Samir et al., 2020).

### 4.2 Distinct TNF Complexes Determine Different Cell Death Signaling Mode

**FIGURE 1:**
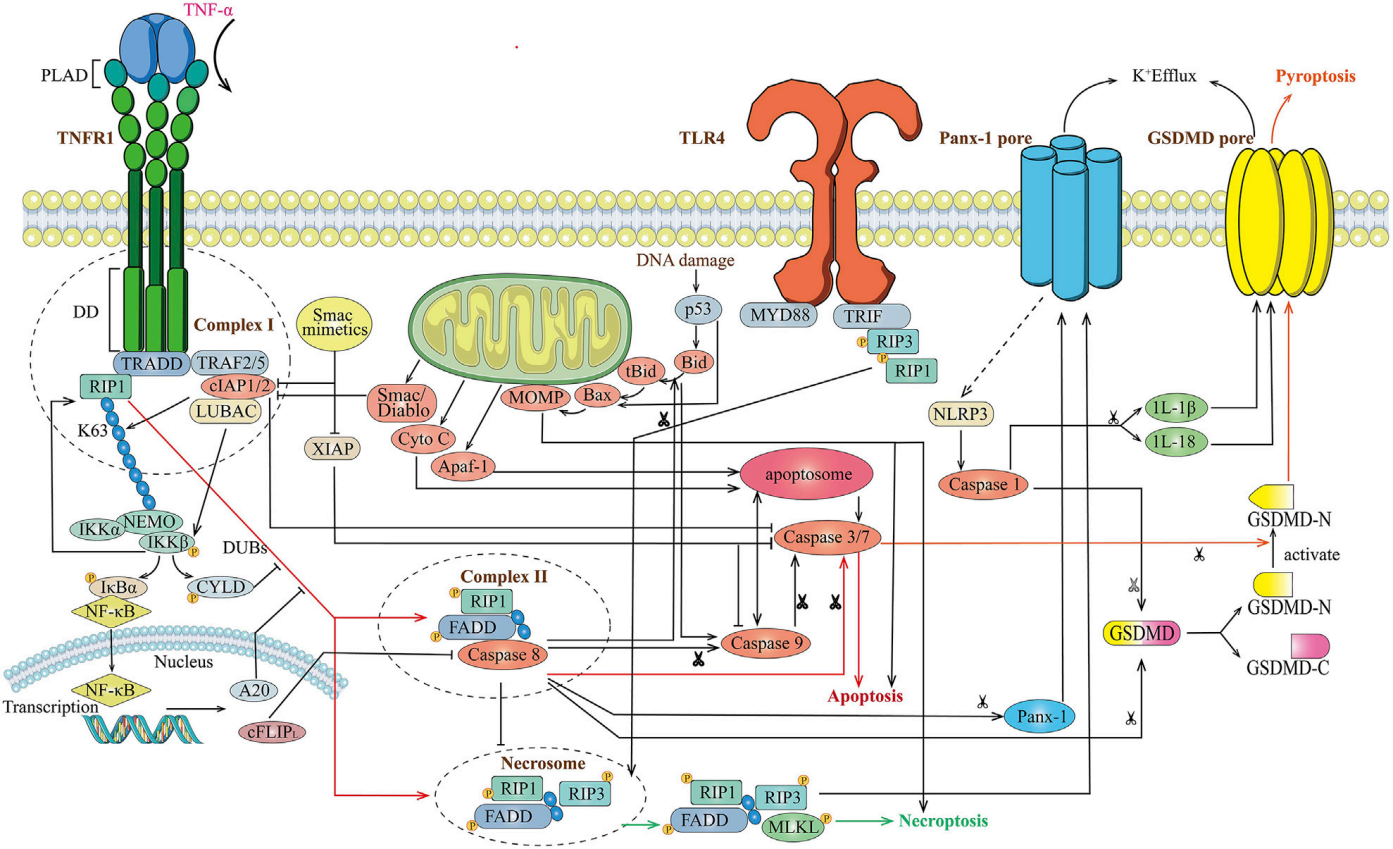
Overview of TNF-induced different cell death signaling mode. Stimulation of cells with TNF leads to recruitment of TRADD and RIP1 to TNFR1, and forms complex I with TRAF2/5, cIAP1/2. The ubiquitination of RIP1 results in IKK-mediated activation of NF-κB and de-ubiquitination of RIP1 results in formation of complex II with FADD and caspase-8, then activates caspase-3 and caspase-7 leading to apoptosis. Meanwhile activated caspase-8 cleaves Gasdermin to form the N-terminal domain (GSDM-N) and C-terminal domain (GSDM-C), which convert apoptosis to pyroptosis. A20 and CYLD block the de-ubiquitination of RIP1 block formation of Complex II and apoptosis. Upon inhibition of caspase-8, RIPK1 activates RIPK3 leading to the formation of the necrosome, which ultimately leads to necroptosis.

The TNF-induced signaling has multiple outcomes: the mode of gene induction, survival, apoptosis, and necroptosis suggesting the existence of different molecule mechanisms of signaling complexes or pathways (Amin et al., 2018) (Figure 1). Firstly, TNFR1 exists in aggregated receptors recruited by the pre-ligand binding assembly domain (PLAD). The formation of complex I was induced by ligand binding owing to a conformational change in TNFR1 that recruits TNF receptor-associated death domain (TRADD), receptor-interacting protein kinase 1 (RIPK1), a cellular inhibitor of apoptosis (cIAPs), TRAF2 (TNFR-associated factor 2)/5, and (linear ubiquitin chain assembly complex) LUBAC protein. The activation of the NF-κB pathway and K63-linked polyubiquitination of RIP1 ultimately constitute the gene induction and survival mode after TNF stimuli. NF-κB translocates to the nucleus and induces transcription, up-regulating A20 and CYLD. RIP1 is targeted by these deubiquitinases for K63 deubiquitination, which balances the strength and activation of NF-κB signaling through negative-feedback mechanisms (Cho et al., 2009; He et al., 2009; Song et al., 2013). A20 and CYLD removes the K63-ubiquitinaiton of RIP1 promoting the transition of membrane-associated complex I (the gene induction or survival mode) to a secondary cytosolic complex II (the apoptosis and necroptosis mode). Two types of complex II related to a formation of necrosome that can ultimately lead to TNF-induced necroptosis are elucidated: TRADD-dependent or RIP1-dependent. After receptor internalization, an RIP-dependent complex of the secondary cytosolic death-initiating signaling complexes (DISC) is formed in the presence of Smac mimetics, which counteract IAPs and lead to apoptosis through the participation of the RIP1 kinase-dependent activation of caspase-8. RIPK1 is essential for Smac mimetic-stimulated TNF-α-induced apoptosis and is also an important molecular switch for the TNF-induced cell death from apoptosis to necrosis (programmed) as RIP3 did (Wang et al., 2008; Zhang et al., 2009). cIAP ablation prevents RIP1 ubiquitination and proteasomal degradation and favors the formation of the complex II without TRADD but a RIP1-FADD scaffold to activate caspase-8 blocking the necroptosis mode. Therefore, the reduced caspase activity can prevent the apoptotic mode and favor the necrotic mode for TNF stimulation because of the presence of caspase inhibitors, or the adaptor molecule FADD, or the absence of caspase-8. When the Smac mimetic was not cotreated in the TNF signaling, TRADD-dependent complex II is formed that RIP1 and RIP3 are recruited, along with Fas-associated death domain protein (FADD) and caspase-8. Proteolytic inactivation of RIP1 and RIP3 will be prevented in the presence of caspase inhibitors, and that sensitizes some cell lines to TNF-induced necrosis. Otherwise, the apoptotic mode was induced with cleavage of RIP1 and RIP3. The formation of these necrosome shares a common characteristic of the absence of caspase-8 highlighting that caspase recruitment may affect the cell death response. The mixed lineage kinase domain-like protein (MLKL) has been identified as necessary for TNF-α-induced necroptosis, which is an interacting partner of RIP3 that jointly compose the necrosome. (Sun et al., 2012). The involvement of RIP1/RIP3 and the MLKL recruitment and phosphorylation finally lead to the induction of programmed necrosis (Das et al., 2016).

The role of TNF-α in cell death continues to be investigated yet the majority of researches focus on its specific role in inducing cell death. In the murine model, virus infection associates an overall increase in the relative levels of cytokines as to the increased innate immune cell lineages of macrophages and neutrophils with a reduction in the percentage of B cells and T cells. (Karki et al., 2021). The combination of TNF-α and IFN-γ can induce high levels of cell death in BMDMs, but limited levels of cell death are found in other cytokine combinations. Accompanying an increased production of TNF-α peak evidenced in patients with virus infection, the synergism of TNF-α and IFN-γ releasing during the disease course induces PANoptosis and perpetuates a cytokine storm to cause tissue (multi-organ) damage and inflammation. (Karki et al., 2021). Cells can experience extensive crosstalk, leading to PANoptosis (pyroptosis, apoptosis, and necroptosis). Furthermore, there is crosstalk between these pathways. Pyroptosis is a caspase-independent cell death with a concomitant inflammatory response and, thus, has been defined as inflammatory cell death pathways with necroptosis. Caspase-3 is a key protein involved in cell apoptosis mode and can also be induced by TNF-α (or chemotherapy drugs) triggering cell pyroptosis; instead, in several cancer cells, caspase-3 cleaves GSDME to generate GSDME-N and GSDME-C activating the N-terminal fragment and causing plasma membrane lysis. (Hu et al., 2020). When high levels of GSDME were expressed, GSDME cleaved by pro-apoptotic caspases convert apoptosis to pyroptosis. Otherwise, apoptosis was induced. Activated caspase-1 and its recruitment cleavages GSDMD into a 30 KD fragment for oligomerization, and the GSDMD N-terminal domain exhibits pro-pyroptotic activity. (Wang et al., 2020). Cells lacking GSDME showed reduced cell death in response to TNFα-induced inflammatory cell death. (Karki et al., 2021). Collectively, GSDMD-mediated loss of membrane integrity results in pyroptosis implicating the main role executing pyroptosis. (Bertheloot et al., 2021). It is generally known that GSDMD-mediated pyroptosis allows a release of the caspase-1 activated cytokines IL-1β and IL-18. (Frank and Vince, 2019). Whether those inflammasome-activated cytokines contribute to necroptosis-induced inflammation remains investigated. Extrinsic apoptosis, a caspase-dependent process, was induced by activating the cysteine protease caspase-8 to form an apoptosome. The essential feature is the release of cytochrome *c* from mitochondria, regulated by the BCL-2 family, initiator caspases (caspase-8, -9, and -10), and effector caspases (caspase-3, -6, and -7). (Bertheloot et al., 2021). In necroptosis, the molecular mechanism of necroptosis is not entirely clear, further underscoring the differences between the animal model and human necroptosis mechanisms. At the leveling of signaling, there are close interplays between apoptosis and necroptosis. A study has suggested that cFLIP_L_ (FLIP) blocks caspase-8-dependent apoptosis, and at the same time, the resulting caspase-8-FLIP heterodimers block RIPK3-dependent signaling requiring for necroptosis in the analysis of FADD, RIPK3 and FLIP, RIPK3 double-knockout mice. (Dillon et al., 2012). High intracellular ATP levels enable a cell to undergo apoptosis, and low ATP level favors necroptosis on the contrary (Eguchi et al., 1997); p53 promotes MOMP to trigger apoptosis and has been recently reported to be required for necroptosis activation (Vaseva et al., 2012); the BCL-2 family of proteins has well-established roles in apoptosis, while their involvement in necroptosis is still under investigation. Current studies have suggested the requirements of the homotypic interactions of RHIMs and the kinase activity of RIPK3 for cell death execution. (Samir et al., 2020).

## 5 Meta-Analysis on the Diagnostic Value of TNF-α Based on 65 Studies

Accordingly, TNF-α plays a prominent role in damaging multi-organs by inducing inflammatory cell death and perpetuating cytokine storm. TNF-α is largely responsible for the timing of iNOS induction by inducing a rapid response. (Karki et al., 2021). Elevated serum TNF-α and mRNA expression in patients with viral myocarditis were consistently observed compared to healthy control implicating the involvement in the pathogenesis of viral myocarditis (Wang et al., 2016; Chen and Deng, 2020). Therefore, we investigated the serum levels of TNF-α, used as serological markers, to identify its VMC values in diagnosis and disease monitoring.

### 5.1 Methods of Meta-Analyses

#### 5.1.1 Search Strategy

Relevant studies were identified through cross-checking of references and by electronic search of databases including PudMed, Embases, Cochrane Library, Scopus, ScienceDirect, and Chinese National Knowledge Infrastructure (CNKI). The following terms were used in the search strategy: (“necroptosis” OR “programmed necrosis” OR “necrotic cell death”), “viral myocarditis,” and “Tumor Necrosis Factor alpha” as well as their abbreviations and synonyms and all possible combination. The detailed search strategy is shown in the supplementary material.

#### 5.1.2 Inclusion and Exclusion Criteria

The studies were included in this analysis if they met the same condition and inclusion criteria as follows: 1) investigation of the TNF-α level and its diagnostic potential for viral myocarditis; and 2) studies provided sufficient data including exact data of the serum TNF-α measurement, sensitivity, and specificity. The exclusion criteria were as follows: 1) studies contributed no data to our analysis; and 2) studies were not in the form of original articles.

#### 5.1.3 Data Extraction and Statistical Analysis

Data were extracted from all eligible studies: the first author’s name, year of publication, number of participants, patient characteristics, age, the expression level of TNF-α, diagnostic accuracy data for VMC detection, the optimal cut-off values, etc. Meta-analysis was carried out by STATA 15.0 software. Continuous data of serum TNF-α expression levels in different periods were summarized using STD mean difference (SMDs) and 95%CIs as their effect sizes and a 95%CI excluding the point of no effect indicates statistical significance. The pooled sensitivity, specificity, and area under the curve (AUC) of the area under the summary receiver operating characteristic (SROC) were estimated to show the diagnostic accuracy of serum TNF-α. Heterogeneity across studies was assessed using the I2 statistic, and an I2 > 50% with a p-value < 0.05 was considered as having significant heterogeneity.

### 5.2 Results

#### 5.2.1 Resutls of Literature Search

We conducted a meta-analysis according to the guidelines set forth by the 2020 PRISM statement and discussed the diagnostic efficiency of serum TNF-α in viral myocarditis. A flow diagram of the study selection is displayed in Supplementary Figure S1. In brief, 702 potentially relevant articles were obtained from electronic databases and then 611 of them were excluded after matching the inclusion/exclusion criteria. Finally, 65 studies met the inclusion criteria and were included in the meta-analysis. Quality assessment was conducted according to the QUADAS-2 tool. In detail, all studies demonstrated a moderate risk of bias among the included studies.

#### 5.2.2 Resutls of Meta-Analyses

During our meta-analysis on the existing literature of the clinical researches, serum TNF-α was significantly increased for the myocarditis group with an SMD of 3.54 (95% CI, 2.99–4.08; p < 0.05; Table 2) compared to controls in its acute stage. A significant decrease of TNF-α in the recovery period was assessed (SMD −3.23, 95% CI −3.89, −2.56; p < 0.05; Table 2) when compared to the acute period. All differences between groups were significant. Significant heterogeneity was observed (I2 >50%, p < 0.05) and a random-effect model was used. Subgroup analysis and sensitivity analysis were also conducted for the assessment of heterogeneity. To account for the potential source of between-study heterogeneity, subgroup analyses were further conducted based on age (children and adults), while no impact of age difference on heterogeneity was found. Both children and adults showed significantly elevated TNF-α levels in the acute stage and decreased TNF-α levels in the recovery stage. When compared to the control group, the VMC group in the recovery stage was still having a significantly higher TNF-α level (SMD 1.41, 95% CI 1.02, 1.81; p < 0.05; Table 2) indicating a possible role for TNF-α during the prognosis period.

**TABLE 2:** Pooled analysis of TNF-α level in comparison to a different stage and subgroup analysis based on age

Comparison	No. of studies	SMD	95% CI	p	I2 (%)	p for heterogeneity
VMC patients vs. control	65	3.6	3.04, 4.15	<0.001	89	0.05
Adult	26	3.40	2.38, 4.41			
Children	39	3.72	3.10, 4.33			
VMC patients in acute vs. recovery stage	32	−3.23	−3.89, -2.56	<0.001	68.6	<0.01
Adult	11	−3.53	−4.71, -2.36			
Children	21	−3.10	−3.94, -2.26			
VMC patients in recovery stage vs. control	32	1.26	0.90, 1.63	<0.001	70.1	0.06
Adult	11	4.48	0.69, 2.28			
Children	21	1.15	0.77, 1.53			

SMD, STD mean difference.

The meta-analyses were calculated from random effect model analysis.

The application of serum TNF-α for identifying acute viral myocarditis patients presented a pooled sensitivity of 79% (95% CI 0.67–0.88) and specificity of 86% (95% CI 0.74–0.93). The pooled positive likelihood ratio (PLR) was 5.7 (95% CI, 3.0–10.6) and the pooled negative likelihood ratio (NLR) was 0.24 (95% CI, 0.15–0.39). Using a random-effects model, the DOR was 24 (95% CI, 11–53). There was no threshold effect, as indicated by Spearman correlation analysis (−0.44; p = 0.19). We used Deek’s funnel plot to evaluate the publication bias of included studies and the shape of the funnel plot showed no between-study heterogeneity (p = 0.19), and no publication bias was found (p = 0.30). The AUC was 0.90 (95% CI 0.87–0.92) implicating that the use of serum TNF-α can provide reliable performance for the diagnosis of VMC (Supplementary Figures 7, 8s).

### 5.3 TNF-α as a Potential Biomarker in VMC

The meta-analysis further confirmed that TNF-α was significantly higher in patients with viral myocarditis and gradually decreased as the disease progressed. Serum TNF-α could be a useful marker for identifying VMC in acute and recovery periods. The virus infection in myocarditis is characterized as a substantial production of TNF-α. Under the strong stimulation of TNF-α, complex II was formed and ultimately caused cardiomyocyte degeneration, necrosis, apoptosis, myocardial interstitial hyperplasia, and an immune-inflammatory response depending on whether caspase-8 is inhibited (Corsten et al., 2012). Overexpression of TNF-α can also confer cytoprotection reducing ischemia-reperfusion (I/R) injury through TRAF2-mediated activation of NF-κB (Burchfield et al., 2010).

## 6 Necroptosis, Cardiomyocyte Death and VMC Progress

During the evaluation of CVB3-infected myocarditis, serum concentrations of creatine kinase, CK-MB, and cardiac troponin I (cTnI) were significantly increased in the CVB3-infected mice than the noninfected group and the model group with Nec-1, suggesting severe damage to cardiomyocytes in the VMC mice models. Histopathologic features also showed distinct changes between groups. In the CVB3 infected mice, necrosis of most cardiomyocytes was observed, as well as myocardial swelling, unclear and strong acidophil staining, pyknosis, karyorrhexis, karyolysis, cell outline disappearance, and inflammatory cell infiltration. Myofilament and myotome were normal under an electronic electroscope of the control group; the cardiac muscle fibers were well-arranged. In the CVB3 group, mitochondrial swelling and severity vacancy-like denaturation with visible infiltrating mononuclear cells and leukomonocyte revealed that necroptosis is involved in viral myocarditis. Necroptosis has been certified to determine a crucial effort on the pathophysiological change of various diseases. Among patients suffering acute critical illnesses such as MI, septic shock, or acute viral myocarditis, the activation of caspase is inhibited due to the inadequacy of adenosine triphosphate (ATP) a cell can produce. Once the activation of caspases (in this case, caspase-8) was prevented, the necrotic signaling is initiated. Necrostatin-1, a necroptosis pathway inhibitor, can dramatically reduce inflammatory infiltration in cardiomyocytes and promote mice survival via blocking the inhibition of RIP1 kinase (He et al., 2009; Zhou et al., 2018). The study of Wu et al. has also implied that necroptosis is related to experimental autoimmune myocarditis (EAM) (Wu et al., 2021).

The current research progress is the application of the human-induced pluripotent stem cells (hiPSCs) in establishing an *in vitro* model of CVB3-induced myocarditis, investigating the molecular mechanisms of viral myocarditis via iCell® Cardiomyocytes (Kraft et al., 2021). It is said to be newly suitable for cardiomyocytes than other cardiac-like cells since cardiomyocytes represent the target cells of enteroviruses in the human heart, and those cardiac-like and non-cardiac cell lines (HeLa cells, HL-1 cells and H9c2 cells, etc.) that have been used frequently to investigate the mechanism can only distantly represent human cardiomyocytes (Claycomb et al., 1998). Not only the change of histopathology indicating the initiation of necroptosis was a key role in the pathology of VMC; the experiment implicates that the inhibition of RIPK1 and RIPK3 resulted in a reduction of viral replication by around half (Kraft et al., 2021), suggesting that the activity of RIPK1/3 may also be crucial for CVB3 replication. However, how RIPK3 functions on CVB3 replication in the cardiomyocytes remains unknown, and the time element should be specially considered about the “paradox” until the exact mechanism is accurately defined.

## 7 Insight From the Relation Between Necroptosis and VMC

**FIGURE 2:**
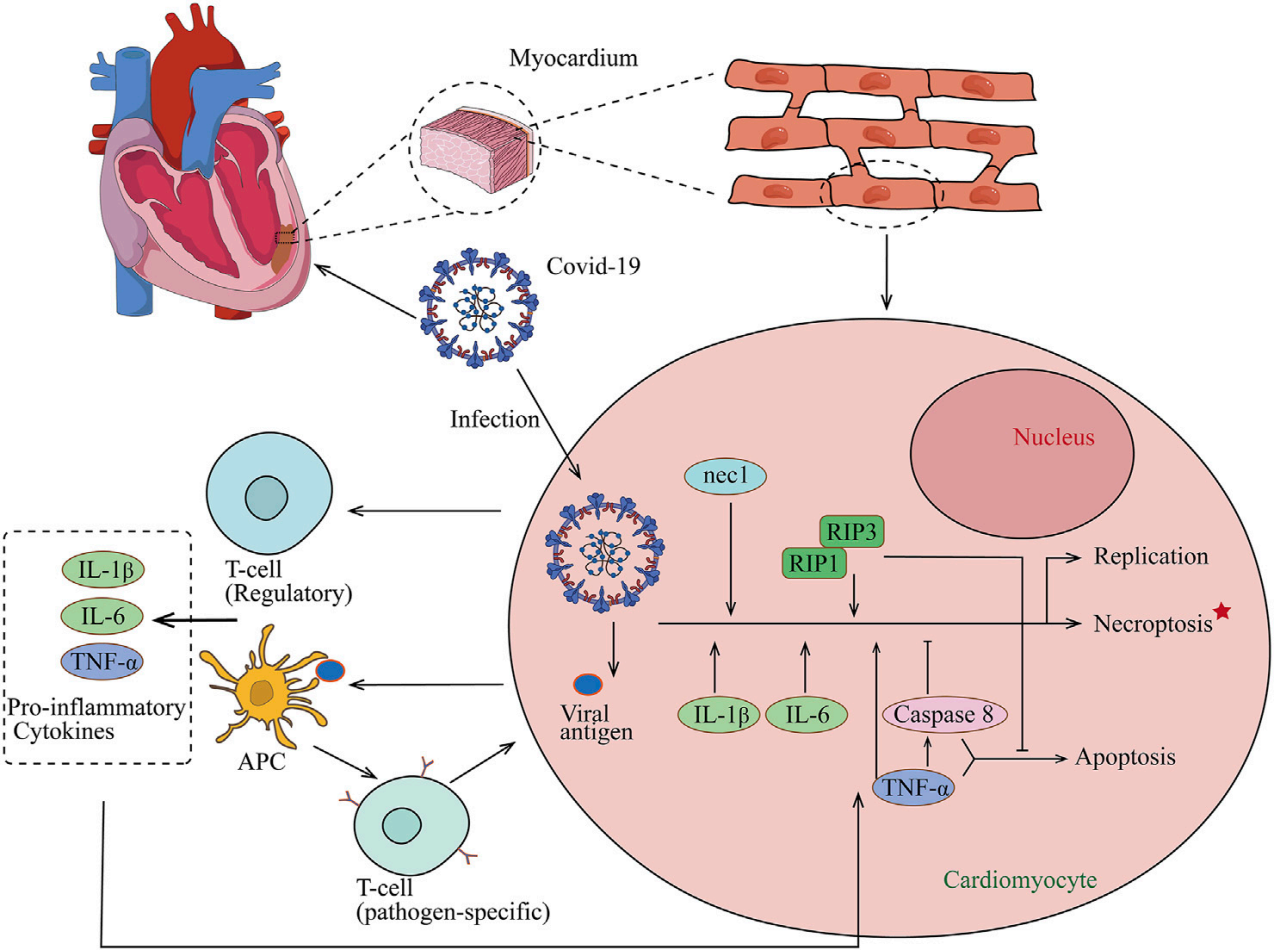
Proposed model for COVID-19 association with myocarditis. After COVID-19 infection, naive T lymphocytes can be primed for viral antigens via antigen-presenting cells, and proinflammatory cytokines of IL-1B, IL-6, and TNF-α which are involved in necroptosis are released into the circulation. The release of cytokines results in a positive feedback loop of immune activation and myocardial damage and also helps the development of COVID-19.

Necroptosis is not equivalent to the concept of necrosis but illustrates a new concept that cell death can be regulated through a series of signal transductions instead of dying passively, therefore suggesting a new therapeutic option, and blocking the necroptosis pathway may serve as a cardiovascular protective role in acute viral myocarditis. Direct evidence has implicated that necroptosis is the significant contributor to myocardial injury in VMC using Nec-1 to block the necroptosis signaling pathway and prevents myocardial injury via downregulating RIP1 and RIP3 expression (Zhou et al., 2018). Necrostatin-1, identified as having the ability to attenuate cell necroptosis in various inflammatory diseases, inhibits overexpression and endogenous RIP1 autophosphorylation (Degterev et al., 2008). The CVB3-induced viral myocarditis model showed significantly stronger RIP1 and RIP3 staining in myocardial tissues compared to the controls and demonstrated disseminated distribution. Nec-1 exerts significant downregulation of positivity indicating the protective effect of Nec-1 in VMC. The protective role of Nec-1 has been reported in various diseases (Yang et al., 2018; Liu et al., 2015). Instead of suppressing autophagy, a cell death process involved in many cellular aspects, Nec-1 can be developed as a potential therapeutic target for inflammatory myocarditis. As of now, infection of coronavirus (COVID-19) causes the quick activation of the immune system and continuous inflammatory response (Figure 2). Cytokines of IL-1B, IL-6, and TNF-α involved in necroptosis have been proven as key cytokines in the development of COVID-19 (Moore and June 2020). Therefore, using Nec-1 may be a potential strategy to treat COVID-19 (Cao and Mu, 2021). In addition, because RIP3 is an energy metabolism regulator switching TNF-induced apoptosis to necroptosis and the key determinant, the deficiency of RIP3 ameliorated myocardial necroptosis and heart failure by inhibiting cardiac remodeling (Cho et al., 2009; Sun et al., 2012; Zhao et al., 2012; Wu et al., 2013; Humphries et al., 2015; Zhang et al., 2016).

## 8 Future Directions

The updated investigation on programmed cell death provides insight into the pathology mechanism of myocarditis. Necroptosis plays an important role in cardiomyocyte death in acute viral myocarditis. Low sensitivity of viral detection and diagnostic methods hinders the treatment for acute myocarditis and may result in the development of dilated cardiomyopathy in partial patients (Pollack et al., 2015). For the establishment of more appropriate treatment strategies, cytokine TNF-α, inducing necroptosis, presents its significant performance for VMC diagnosis and can be used as a biomarker for subsequent prognosis prediction. Recent researches in the relationship between necroptosis and cardiovascular diseases are still in its beginning stage. While CVB3-induced viral myocarditis is the predominant model for viral myocarditis exploration, other types of viruses associated with inflammation or their relationship with other forms of cell death remains an effort to explore. Currently, researches on necroptosis are insufficient, for example, the roles of death receptor signaling and crosstalk between necrosis, apoptosis, and necroptosis make differentiation difficult to define *in vivo*. Also, the role of necroptosis in diseases across the body including virus induced-cancer is warranted. Optimal window period and markers are poorly defined when marking cell death. Identifying these unknown mechanisms and biomarkers will lead to a novel understanding of necroptosis and new therapeutic treatments for their related diseases. The application of Nec-1 is worth investigating as it can not only contribute to the comprehension of necroptosis but also contribute to identifying the potential therapeutic targets.
