# Supplementary material for: Necroptosis and Viral Myocarditis: Tumor Necrosis Factor α as a Novel Biomarker for the Diagnosis of Viral Myocarditis
Source: Front Cell Dev Biol. 2022 May 4;10:826904. doi: 10.3389/fcell.2022.826904 (PMC9114881; doi:10.3389/fcell.2022.826904)
Supplement: Supplementary file 1 [file DataSheet1.docx]

**Supplementary Appendix**

Supplement to: Necroptosis and viral myocarditis: tumor necrosis factor α as a novel biomarker for the diagnosis of viral myocarditis

Contents

[Meta-analysis on the relationship between serum TNF-alpha and viral myocarditis 1](#_Toc87864865)

[Search strategy 1](#_Toc87864866)

[*1.1.* *PubMed search strategy* 1](#_Toc87864867)

[*1.2.* *Embases search strategy* 1](#_Toc87864868)

[*1.3.* *Cochrane Library search strategy* 1](#_Toc87864869)

[*1.4.* *Scopus search strategy* 2](#_Toc87864870)

[*1.5.* *Science Direct search strategy* 2](#_Toc87864871)

[*1.6.* *CNKI search strategy* 2](#_Toc87864872)

[Supplementary figures 3](#_Toc87864873)

[Figure 1 Flow diagram of literature selection 3](#_Toc87864874)

[Figure 2 Risk of bias of the 65 included studies (red: high; yellow: unclear; green: low) 3](#_Toc87864875)

[Figure 3 Forest plot of pooled serum TNF-α between viral myocarditis and control 4](#_Toc87864876)

[Figure 4 Subgroup analysis of pooled serum TNF-α between viral myocarditis and control 5](#_Toc87864877)

[Figure 5 Forest plot of pooled serum TNF-α between viral myocarditis in acute and recovery stage 6](#_Toc87864878)

[Figure 6 Forest plot of pooled serum TNF-α between viral myocarditis in recovery stage and control 7](#_Toc87864879)

[Figure 7 Diagnostic accuracy of serum TNF-α in diagnosis viral myocarditis 8](#_Toc87864880)

[Figure 8 SORC for the diagnostic accuracy of serum TNF-α in diagnosis viral myocarditis 9](#_Toc87864881)

[Included studies 10](#_Toc87864882)

# Meta-analysis on the relationship between serum TNF-alpha and viral myocarditis

## Search strategy

### PubMed search strategy

Pubmed was searched on 23/10/21 without language limitation.

1. (“Tumor Necrosis Factor alpha” [tiab] OR “Cachectin” [tiab] OR “Cachectin-Tumor Necrosis Factor” [tiab] OR “Cachectin Tumor Necrosis Factor” [tiab] OR “Tumor Necrosis Factor Ligand Superfamily Member 2” [tiab] OR “Tumor Necrosis Factor” [tiab] OR “TNF Superfamily, Member 2” [tiab] OR “TNFalpha” [tiab] OR “TNF-alpha” [tiab] OR “TNFα” [tiab] OR “TNF-α” [tiab] OR“TNF” [tiab])
2. (“viral myocarditis” [tiab] OR "Myocarditides"[tiab] OR "myocarditis"[tiab])
3. #1 AND #2

Key: tiab= title or abstract

Result 126

### Embases search strategy

The database of web of science was searched on 20/09/21 without language limitation.

TS = 1 (“Tumor Necrosis Factor alpha” OR “Cachectin” OR “Cachectin-Tumor Necrosis Factor” OR “Cachectin Tumor Necrosis Factor” OR “Tumor Necrosis Factor Ligand Superfamily Member 2” OR “Tumor Necrosis Factor” OR “TNF Superfamily, Member 2” OR “TNFalpha” OR “TNF-alpha” OR “TNFα” OR “TNF-α” OR “TNF” ) AND (“viral myocarditis” OR "Myocarditides" OR "myocarditis")

Key: TS= topic, abstract, keyword

Result 112

### Cochrane Library search strategy

Cochrane Library was searched on 23/09/21 without language limitation.

| Search | Query | Results |
| --- | --- | --- |
| #1 | "myocarditis".ti,ab,kw. | 1,204 |
| #2 | "Myocarditides".ti,ab,kw. | 0 |
| #3 | "Carditis".ti,ab,kw. | 45 |
| #4 | 1 OR 2 OR 3 | 1,244 |
| #5 | "Tumor Necrosis Factor-alpha".ti,ab,kw. | 4,706 |
| #6 | "Cachectin".ti,ab,kw. | 5 |
| #7 | "Cachectin Tumor Necrosis Factor".ti,ab,kw. | 3 |
| #8 | "Tumor Necrosis Factor".ti,ab,kw. | 8,631 |
| #9 | "TNFalpha".ti,ab,kw. | 1,573 |
| #10 | "TNF-alpha".ti,ab,kw. | 7,682 |
| #11 | "TNF-α".ti,ab,kw. | 13,398 |
| #12 | "TNFα".ti,ab,kw. | 13,398 |
| #13 | 5 OR 6 OR 7 OR 8 OR 9 OR 10 OR 11 OR 12 | 17,856 |
| #14 | 4 AND 13 | 3 |

ti = title, ab = abstract, kw = keyword.

### Scopus search strategy

| Search | Query | Results |
| --- | --- | --- |
| #1 | "myocarditis" | 16,744 |
| #2 | "Myocarditides" | 12 |
| #3 | "Carditis" | 1,231 |
| #4 | #1 OR #2 OR #3 | 17,844 |
| #5 | "Tumor Necrosis Factor-alpha" | 76,186 |
| #6 | "Cachectin" | 2,497 |
| #7 | "Cachectin Tumor Necrosis Factor" | 69 |
| #8 | "Tumor Necrosis Factor" | 182,176 |
| #9 | "TNFalpha" | 3,460 |
| #10 | "TNF-alpha" | 177,932 |
| #11 | "TNF-α" | 0 |
| #12 | "TNFα" | 0 |
| #13 | #5 OR #6 OR #7 OR #8 OR #9 OR #10 OR #11 OR #12 | 284,736 |
| #14 | #4 AND #13 | 290 |

### Science Direct search strategy

The database of Science Direct was searched on 30/08/21 without language limitation.

| Search | Query | Results |
| --- | --- | --- |
| #1 | ("myocarditis" OR "Myocarditides" OR "Carditis") AND ("Tumor Necrosis Factor-alpha" OR "Cachectin" OR "Tumor Necrosis Factor" OR "TNFalpha" OR "TNF-alpha") | 10 |

### CNKI search strategy

The database of Medline was searched on 30/08/21 without language limitation.

| Search | Query | Results |
| --- | --- | --- |
| #1 | Key word:肿瘤坏死因子 (TNF) AND Key word:心肌炎 (myocarditis) | 179 |

## Supplementary figures

## Figure 1 Flow diagram of literature selection


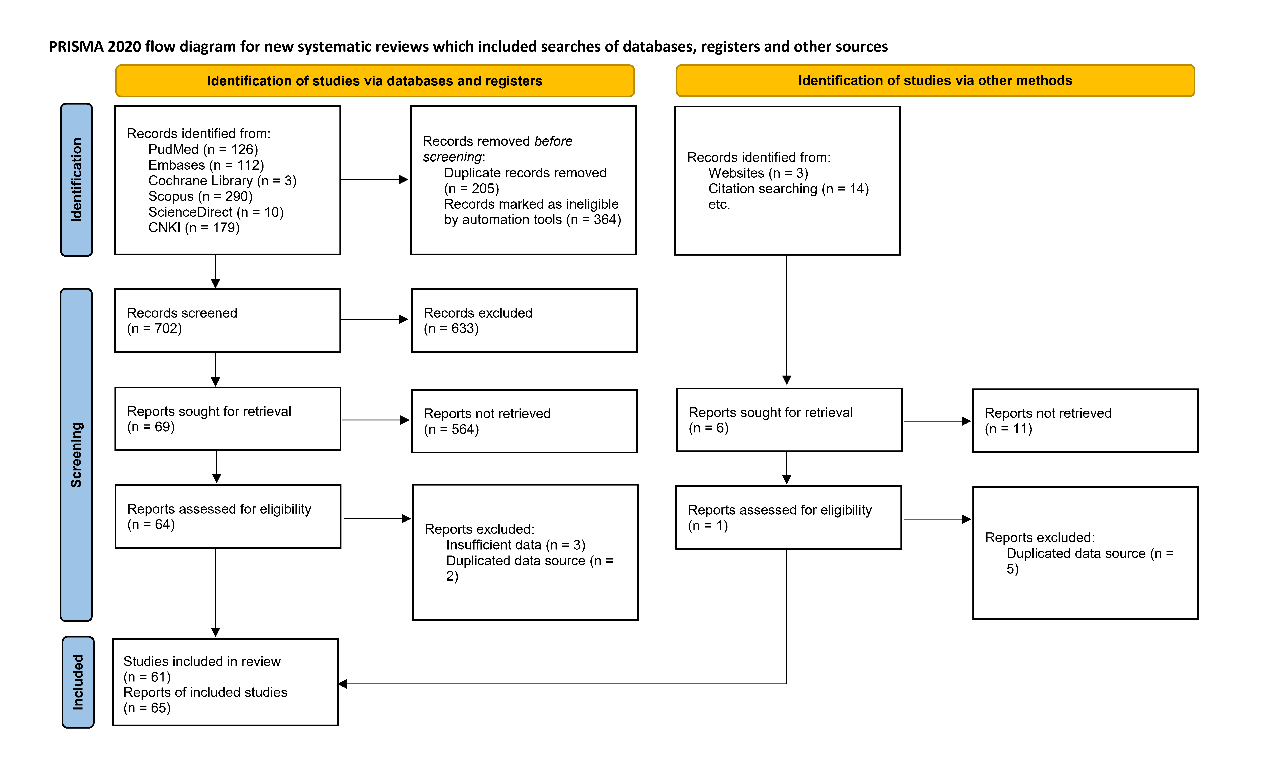


## Figure 2 Risk of bias of the 65 included studies (red: high; yellow: unclear; green: low)


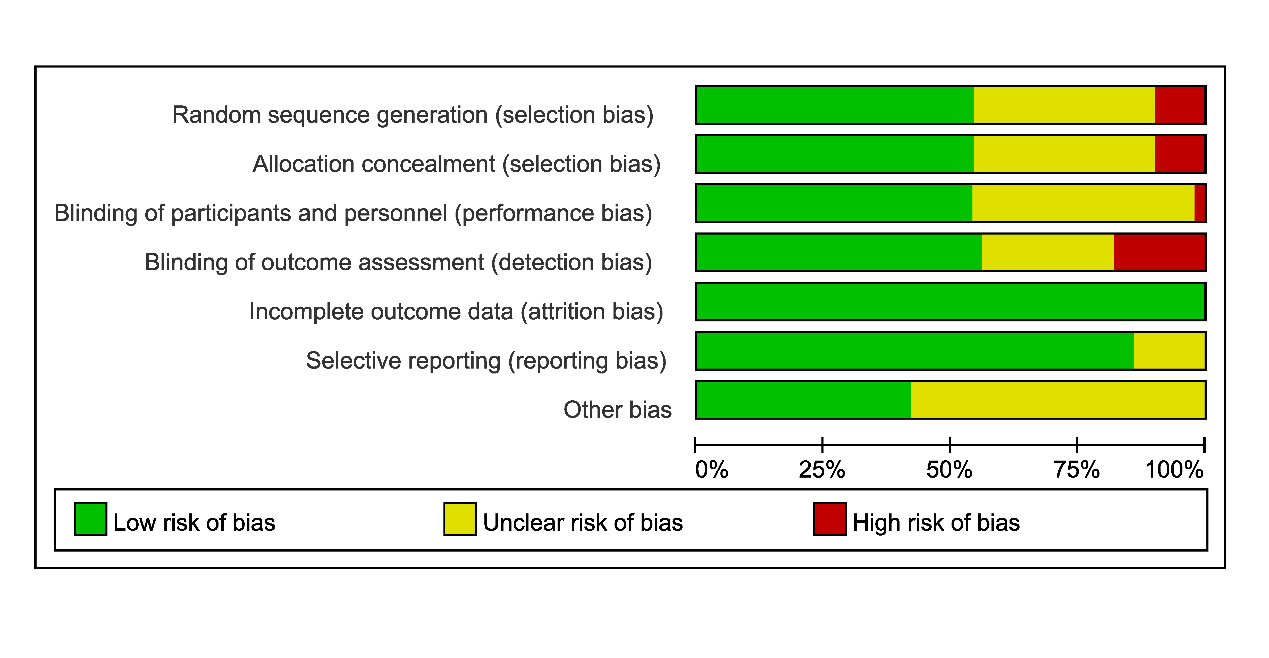


## Figure 3 Forest plot of pooled serum TNF-α between viral myocarditis and control


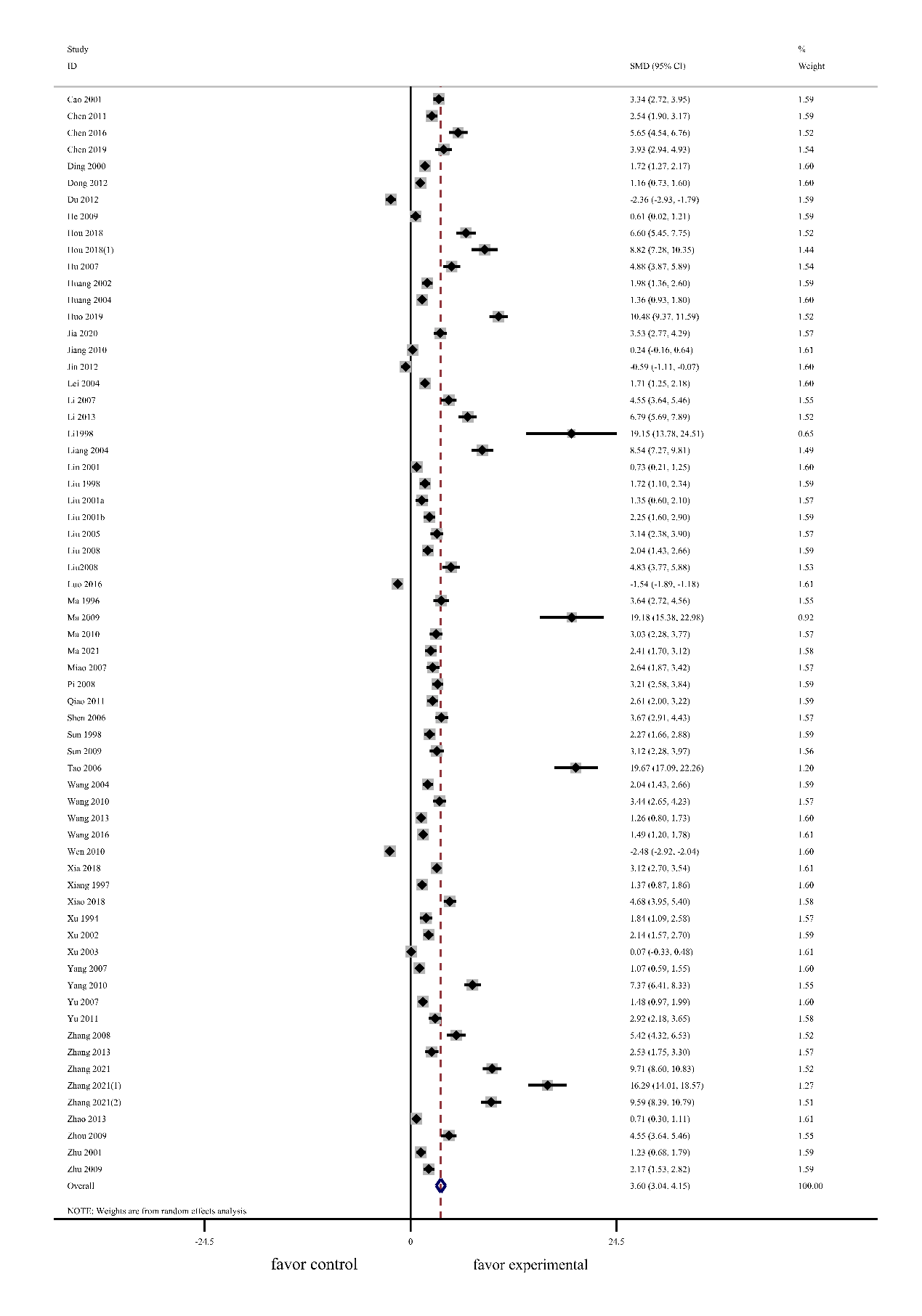


## Figure 4 Subgroup analysis of pooled serum TNF-α between viral myocarditis and control


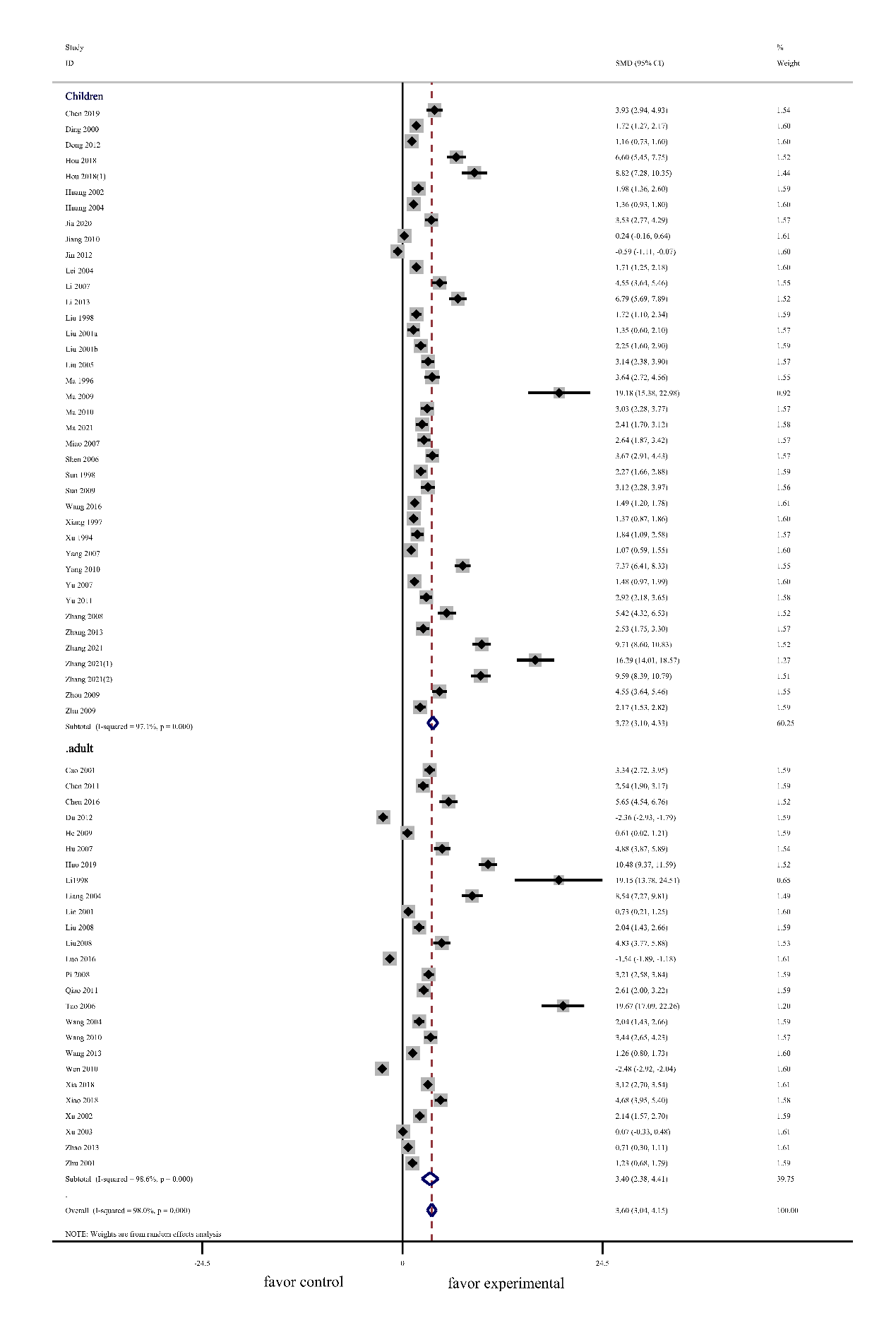


## Figure 5 Forest plot of pooled serum TNF-α between viral myocarditis in acute and recovery stage


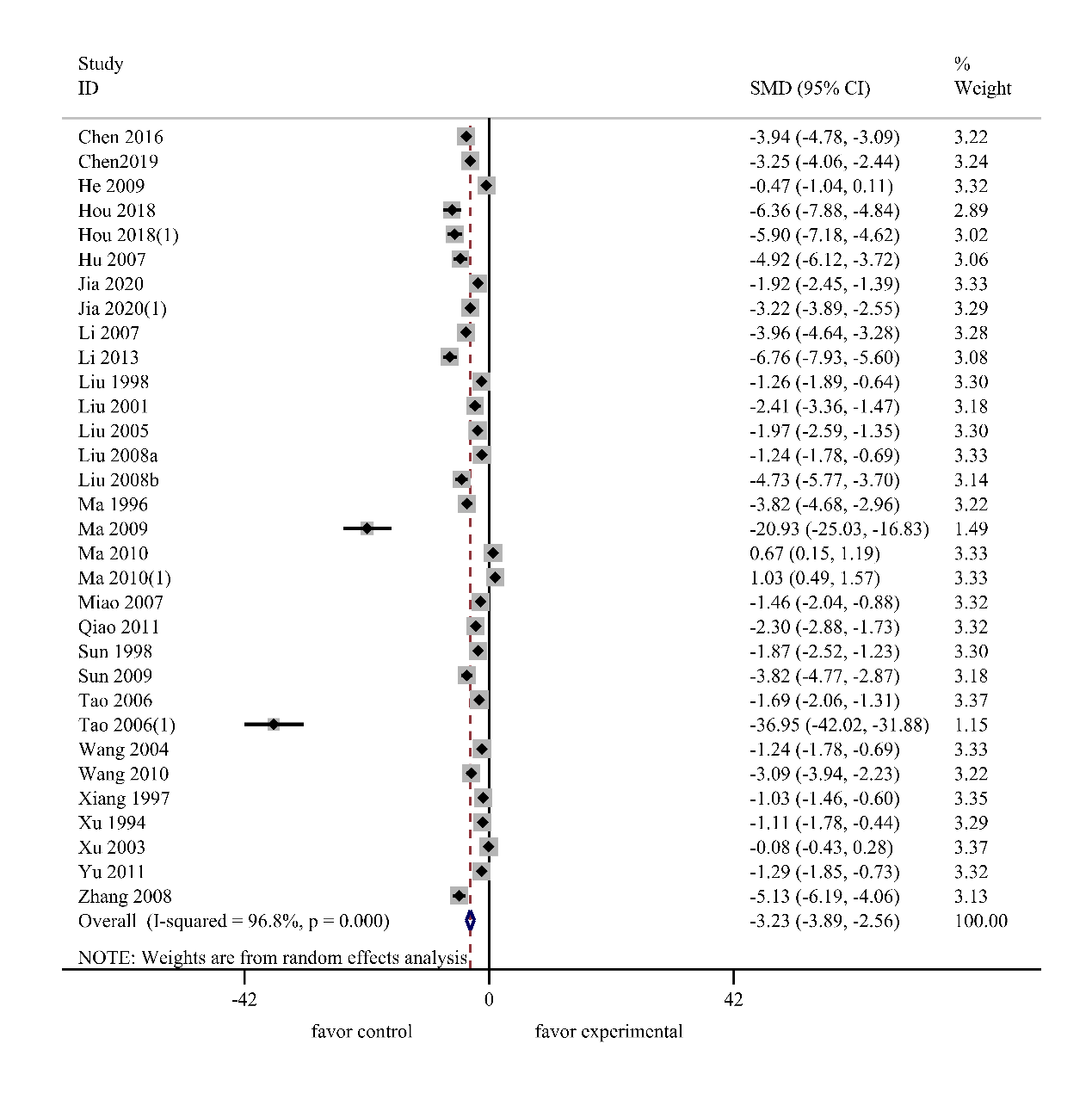


## Figure 6 Forest plot of pooled serum TNF-α between viral myocarditis in recovery stage and control


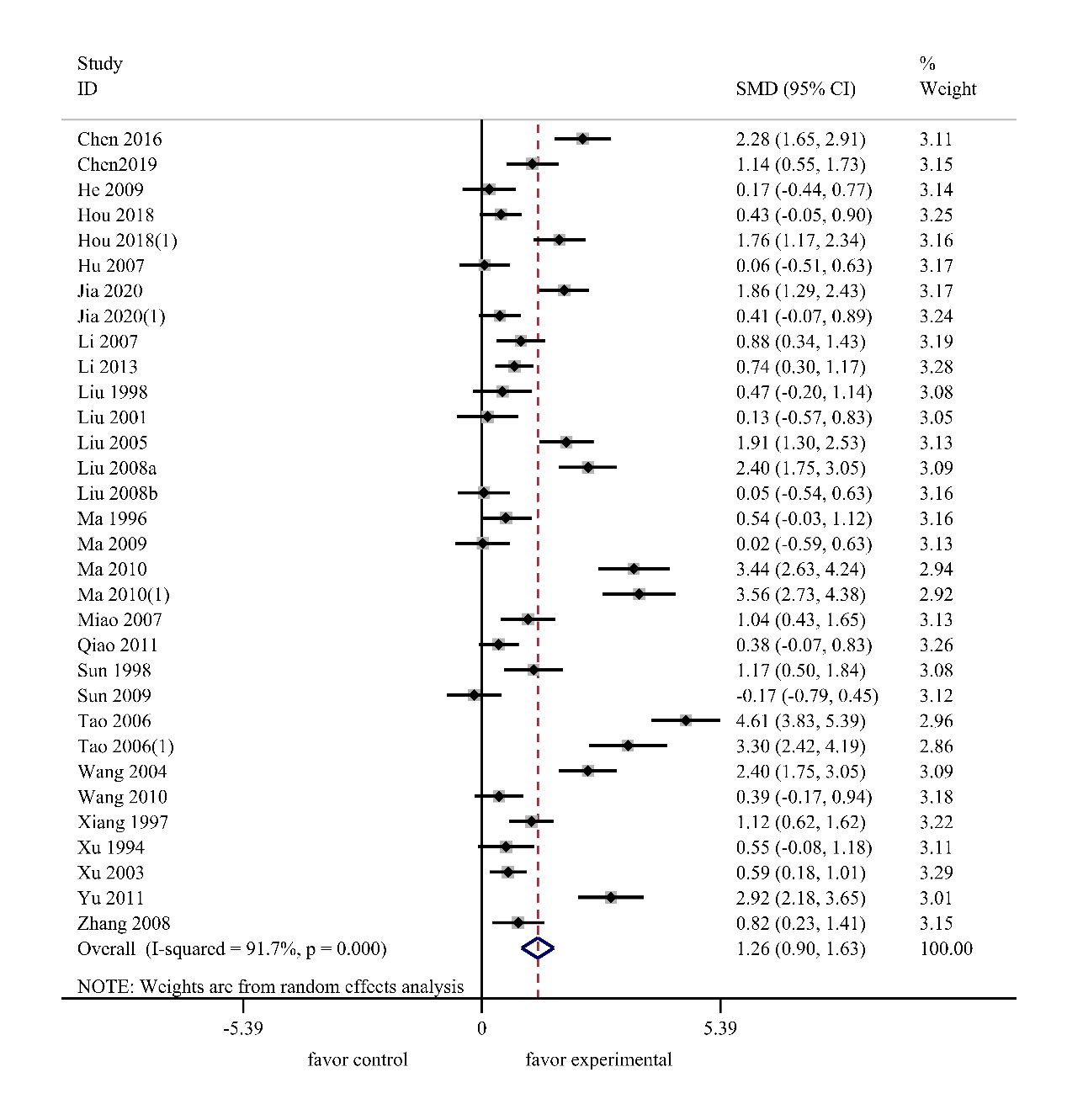


## Figure 7 Diagnostic accuracy of serum TNF-α in diagnosis viral myocarditis


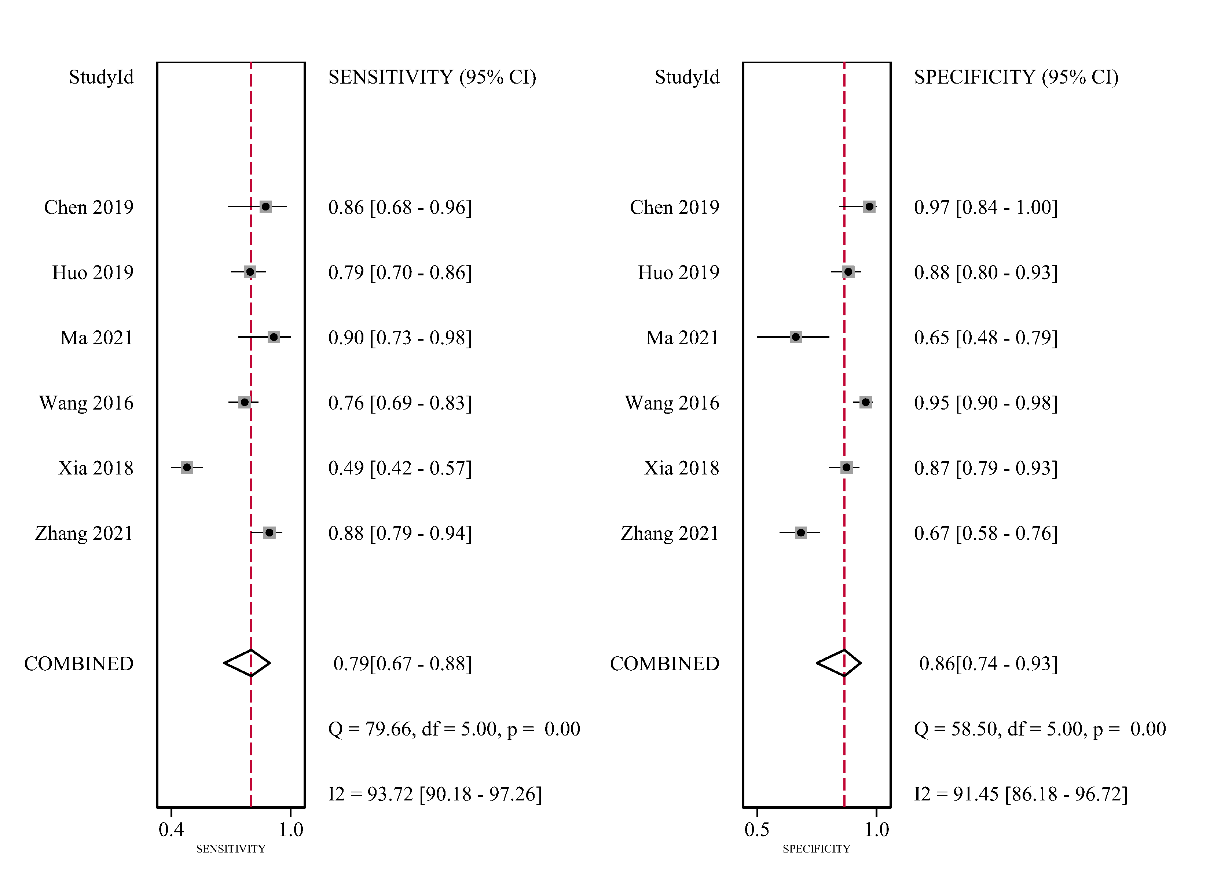


## Figure 8 SORC for the diagnostic accuracy of serum TNF-α in diagnosis viral myocarditis


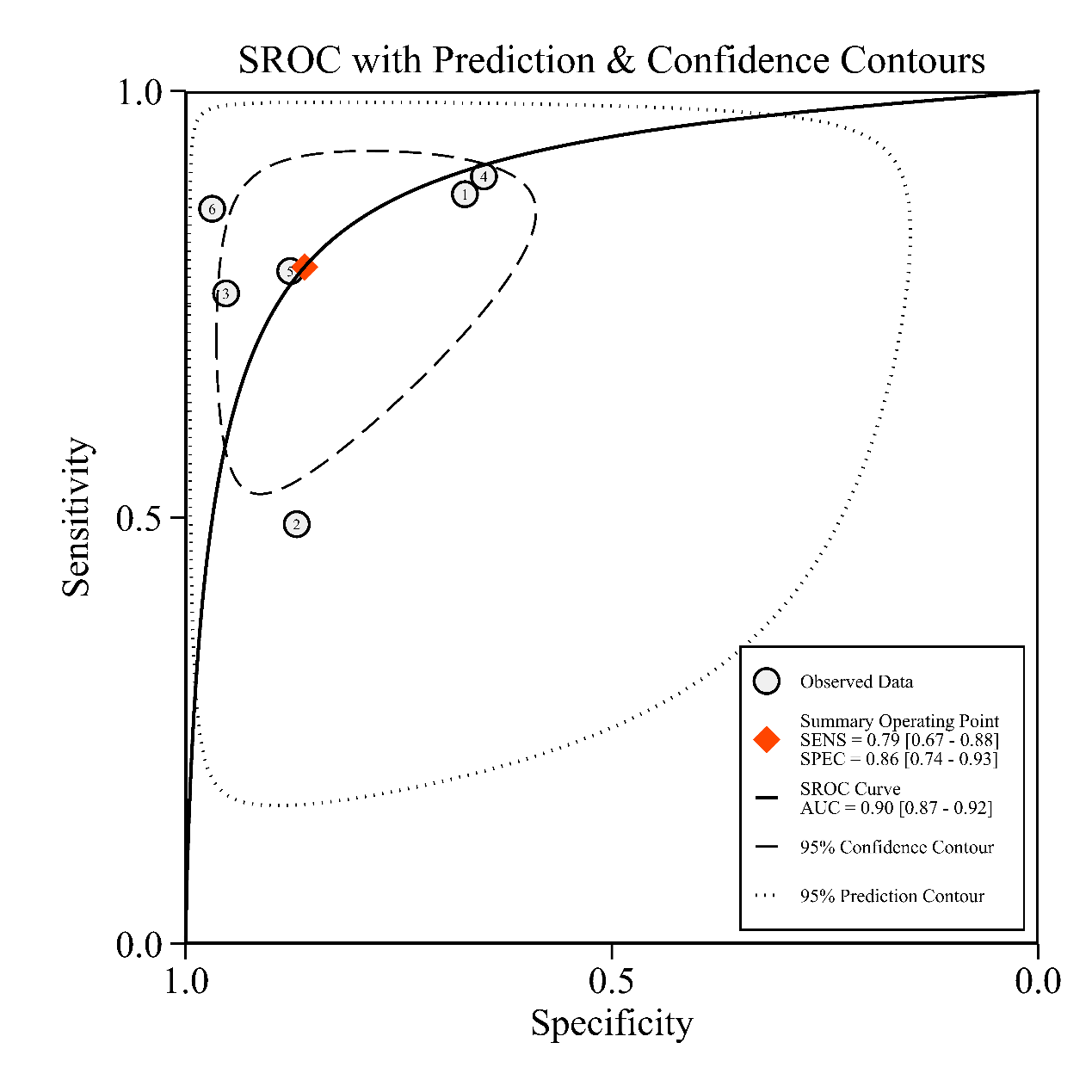


## Included studies

**^1-61^**

1. Zhang S, Jing N. Application value of myocardial enzymes combined with hs-CRP and TNF-α detection in the diagnosis and severity evaluation of viral myocarditis. *Hainan Med J*. 2021;32(9):1144-1147. doi:10.3969/j.issn.1003-6350.2021.09.015

2. Ma W, Li S. Analysis of serum TNF-α,CK-MB,cTnI level detection in diagnosis of children with viral myocarditis. *CHINESE COMMUNITY DOCTORS*. 2021;37(5):110-111. doi:10.3969/j.issn.1007-614x.2021.05.053

3. Jia F, Zhang H. Relationship between serum TNF-α and NF-κB levels and onset of disease in children with viral myocarditis. *Chinese Remedies & Clinics*. 2020;20(12):1943-1945. doi:10.11655/zgywylc2020.12.007

4. Huo M, Zhang N, Hu N, Sun L, Wang H. The Value Evaluation of Serum miR-1 Combined with cTn Ⅰ and TNF-α in the Diagnosis and Prognosis of Viral Myocarditis. *标记免疫分析与临床*. 2019;26(7):1190-1195. doi:10.11748/bjmy.issn.1006-1703.2019.07.026

5. Chen J, Deng Y. Diagnostic Performance of Serum CK-MB, TNF-α and Hs-CRP in Children with Viral Myocarditis. *Open life sciences*. Jan 2019;14:38-42. doi:10.1515/biol-2019-0005

6. Xiao Y, Yuan L, Ding B, et al. Research for correlation among echocardiography indexes and immune function in patients with viral myocarditis. *Chin J Cardiovasc Rehabil M ed* 2018;27(4):437-440. doi:10.3969/j.issn.1008-0074.2018.04.18

7. Xia W. Diagnostic and Prognostic Values of Serum cTn Ⅰ, MIF and TNF-α in Viral Myocarditis. *Labeled Immunoassays & Clin Med*. 2018;25(8):1154-1158. doi:10.11748/bjmy.issn.1006-1703.2018.08.018

8. Hou W, Wang F, Feng Y. Expression and clinical significance of serum miR-1 and miR-146b in children with viral myocarditis. *J Trop Med*. 2018;18(5):629-633. doi:10.3969/j.issn.1672-3619.2018.05.018

9. Luo Z. Changes of cellular immune function in patients with viral myocarditis and its clinical application value. *China Prac Med*. 2016;11(30):40-41. doi:10.14163/j.cnki.11-5547/r.2016.30.021

10. Chen R, Chen Y, Jia J, et al. Analysis on TNF-α, TGF-β1 and IFN-γ cytokines levels in peripheral blood of viral myocarditis patients. *Int j Lab Med*. 2016;37(8):1119-1120. doi:10.3969/j.issn.1673-4130.2016.08.045

11. Wang D, Li T, Cui H, Zhang Y. Analysis of the Indicating Value of Cardiac Troponin I, Tumor Necrosis Factor-alpha, Interleukin-18, Mir-1 and Mir-146b for Viral Myocarditis among Children. *Cellular Physiology and Biochemistry*. 01 Dec 2016;40(6):1325-1333. doi:<http://dx.doi.org/10.1159/000453185>

12. Zhang Y, Shi B, Wang Y, Huang K, Huang K. Analysis on levels of TNF-α,TGF-β1,and IFN-γ in peripheral blood of children with viral myocarditis. *Maternal and Child Health Care of China*. 2013;(5):789-790.

13. Wang J, Adijiangjiamali., Liu J. Analysis of Cytokines and T-Cell Subsets in Patients with Viral Myocarditis. *Clinical Medical & Engineering*. 2013;20(9):1079-1080. doi:10.3969/j.issn.1674-4659.2013.09.1079

14. Li Y, Zhou B, Qin L, et al. The clinical significance of detecting serum levels of TNF-α, hs-CRP and cTnI in patients with viral myocarditis. *Journal of Clinical and Experimental Medicine*. 2013;12(17):1368-1369. doi:10.3969/j.issn.1671-4695.2013.17.012

15. Jin Y. The expression level and clinical significance of serum IL-10, MIF and TNF - α in viral myocarditis children. *Chin J Pract Med*. 2012;39(1):85-86. doi:10.3760/cma.j.issn.1674-4756.2012.01.041

16. Du S. Analysis cellular immune function in the diagnosis and treatment of patients with viral myocarditis. *World Health Digest*. 2012;(48):153-154. doi:10.3969/j.issn.1672-5085.2012.48.142

17. Dong G, Zhao N. Changes of serum cytokines in children with viral myocarditis induced by coxsackie virus type B and its clinical significance. *CHIN J Postgrad Med*. 2012;35(3):14-16. doi:10.3760/cma.j.issn.1673-4904.2012.03.006

18. Yu S, Zhu H. Association between viral myocarditis and inflammatory factors. *Inner Mongolia Medical Journal*. 2011;43(9):1057-1058. doi:10.3969/j.issn.1004-0951.2011.09.012

19. Qiao J. Analysis of serum IL-18, IL-6 and TNF-α levels in 80 cases of viral myocarditis. *Inner Mongolia Medical Journal*. 2011;30(23):81-82. doi:10.3969/j.issn.1006-0979.2011.23.092

20. Chen S. Analysis on the changes of serum IL-18 and TNF-α levels in patients with viral myocarditis. *Practical Journal of Cardiac Cerebral Pneumal and Vascular Disease*. 2011;19(6):930-931. doi:10.3969/j.issn.1008-5971.2011.06.028

21. Yang L, Zhang W, Zhou W. Expressive Significance of Tumour Necrosis Factor-α and Resoluble Receptor Proteinum 5500 in Serum on Child with Viral Myocarditis. *Chinese journal of Medicinal Guide*. 2010;12(4):644,646. doi:10.3969/j.issn.1009-0959.2010.04.064

22. Wen H, Jia W, Liu L, et al. Clinical significance of cellular immune function in patients with viral vocarditis. *Chin J Cell Mol Immunol*. 2010;26(7):685-686.

23. Wang Z, Zhou R, Fang Y, Kuang Z. Changes and Significance of Serum IL - 18, IL - 6 and TNF - α in the Patients with Viral Myocarditis. *Practical Journal of Cardiac Cerebral Pneumal and Vascular Disease*. 2010;18(11):1563-1564. doi:10.3969/j.issn.1008-5971.2010.11.002

24. Ma H. IGF-1 and TNF-αin children with viral expression. *China Modern Medicine*. 2010;17(15):27-28. doi:10.3969/j.issn.1674-4721.2010.15.015

25. Jiang D, Zhou S, Zhou H, et al. Cytokine IL-6, IL-8-8, TNF-α and viral myocarditis. *chin Heart J* 2010;22(2):306-307.

26. Wen HY, Jia WK, Liu L, et al. [Clinical significance of cellular immune function in patients with viral myocarditis]. [Chinese]. *Xi bao yu fen zi mian yi xue za zhi = Chinese journal of cellular and molecular immunology*. Jul 2010;26(7):685-686.

27. Zhu B. Change and significance of TNF-α,IL-6 and IL-8 in child patients with viral myocarditis. *Chongqing Medicine*. 2009;38(2):163,166. doi:10.3969/j.issn.1671-8348.2009.02.016

28. Zhou W. Clinical value of changes of tumor necrosis factor-a and interleukin-6 level in the children with acute viral myocarditis. *Chinese journal of Cardiovascular Research*. 2009;7(4):285-287. doi:10.3969/j.issn.1672-5301.2009.04.016

29. Sun Z, Sun J, Yang M. Analysis on the relationship between neonatal viral myocarditis and the levels of IL-6 and TNF-α. *Maternal & Child Health Care of China*. 2009;24(8):1082-1083.

30. Ma S, Zhang L, Li X. Study of Macrophage Migration Inhibitory Factor, Tumor Necrosis Factor-α and Interferon-γ in Children with Acute Viral Myocarditis. *Chinese Journal of General Practice*. 2009;7(9):927-928.

31. He J, Peng X. Variations and sisniflcance of levels of TNF-а,IL-6 and IL-8 in serum of viral myocarditis patients. *China Tropical Medicine*. 2009;9(6):1042-1043.

32. Zhang X, Yu X, Guo F, Li S. Expressions of Macrophage Migration Inhibitory Factor, Tumor Necrosis Factor-α and Interleukin-1β in Serum of Children with Viral Myocarditis. *J Appl Clin Pediatr*. 2008;23(1):35-36. doi:10.3969/j.issn.1003-515X.2008.01.011

33. Pi Z. Determination of serum nitric oxide, IL-1β and tumor necrosis factor in patients with viral myocarditis and its significance. *Chongqing Medicine*. 2008;37(11):1258-1259. doi:10.3969/j.issn.1671-8348.2008.11.061

34. Liu X, Wang Y, Yu Y. Dynamic change of serum cytokines levels in patients with virus myocarditis and its clinical value. *Modern Medicine Journal of China*. 2008;10(7):50-52. doi:10.3969/j.issn.1672-9463.2008.07.016

35. Liu J, Zhang Y. Detection and significance of tumor necrosis factor-α in viral myocarditis. *China Medical Herald* 2008;5(26):74-74. doi:10.3969/j.issn.1673-7210.2008.26.045

36. Yu X, Sun J, Lv M, Zhang J. Determination the serum of tumor necrosis factor alpha and interleukin 10 in patient with viral myocarditis. *Chinese Journal of Laboratory Diagnosis*. 2007;11(10):1317-1318. doi:10.3969/j.issn.1007-4287.2007.10.015

37. Yang N, Zhang H, Wang J. Study of Serum Interleukin-18,Tumor Necrosis Factor-Alpha,Interferon-Gama Level and Their Relationships in Children with Viral Myocarditis. *Chinese Journal of Applied Clinical Pediatrics*. 2007;22(13):990-991. doi:10.3969/j.issn.1003-515X.2007.13.014

38. Miao X, Li J. Correlation analysis of TNFα, IL-6 and myocardial enzymes in children with myocarditis. *Journal of Changzhi Medical College*. 2007;21(6):447-449. doi:10.3969/j.issn.1006-0588.2007.06.021

39. Li Y, Zhou W, Luan B. Significance of Changes of Tumor Necrosis Factor-α and Interleukin-6 Level in Children with Acute Viral Myocarditis. *Chinese Journal of Applied Clinical Pediatrics*. 2007;22(13):984-985. doi:10.3969/j.issn.1003-515X.2007.13.011

40. Hu B, Xu J, Hong G, Wu Z, Li Z. Detection of three kinds of cytokines in patients with viral myocarditis. *Chinese Journal of Practical Internal Medicine*. 2007;27(5):363-364. doi:10.3969/j.issn.1005-2194.2007.05.016

41. Tao Q, Zhou X, Li E. The Value of Determination of Blood Cardiac Troponin T, IL-8 and TNF-α Contents for the Diagnosis and Prognosis Assessment in Patients with Viral Myocarditis. *J of Radioimmunology*. 2006;19(4):303-304. doi:10.3969/j.issn.1008-9810.2006.04.027

42. Shen J, Ma X. Detection of serum inflammatory cytokines in children with viral myocarditis and its clinical value. *Zhejiang Clinical Medical Journal*. 2006;(12):1324.

43. Liu H, Li J, Wang Y, Zhao X. The relationship between the levels of TNF-a and IL-6 and CK-MB in children with viral myocarditis. *Chinese Journal of Disdiagnostics*. 2005;5(11):2042-2043. doi:10.3969/j.issn.1009-6647.2005.11.024

44. 王焱Wang Y, 王卉呈Wang H, 路非平Lu F, 杜平Du P. The study of relationship between virus myocarditis and tumor necrosis factor. *Journal of Xinjiang Medical University*. 2004;27(2):138-139. doi:10.3969/j.issn.1009-5551.2004.02.014

45. Liang W, Li G, Chen J. Changes of plasma nitric oxide and tumor necrosis factor levels in patients with viral myocarditis. *Chin J Cardiovasc Rehabil Med*. 2004;13(4):333-334. doi:10.3969/j.issn.1008-0074.2004.04.011

46. Lei H. Detection and significance of plasma cytokines in patients with acute viral myocarditis. *Journal of Medical Forum*. 2004;25(7):37,39. doi:10.3969/j.issn.1672-3422.2004.07.020

47. Huang L, Ma P, Wang J, Han X. Changes and significance of interferon-γ and TNF-α in viral myocarditis. *Journal of Clinical Pediatrics*. 2004;22(3):147-148. doi:10.3969/j.issn.1000-3606.2004.03.006

48. Xu J. Level of TNFα in Viral Myocarditis and Its Relationship with Serum CK and cTNT. *Chinese Journal of Disdiagnostics*. 2003;3(10):1543-1543. doi:10.3969/j.issn.1009-6647.2003.10.067

49. Xu Z, Pan X, Lu Q, et al. The clinical significance of cTnT and TNF-αin acute stage of viral myocarditis. *J Clin Cardiol*. 2002;18(3):98-99. doi:10.3969/j.issn.1001-1439.2002.03.002

50. Huang W, Huang H, Wei Y, Wei Z, Pan H. Detection of cytokines in acute stage of viral myocarditis in children and its clinical significance. *Journal of Guangxi Medical University*. 2002;19(2):221-222. doi:10.3969/j.issn.1005-930X.2002.02.031

51. Zhu C, Zhang J, Yang M, Ding B. CHANGES OF TNF, sIL-2R, IL-6, IL-8 LEVELS, T-LYMPHOCYTE SUBSETS AND THEIR CLINICAL SIGNIFICANCES IN PATIENTS WITH VIRAL MYOCARDITIS OR IDIOPATHIC DILATED CARDIOMYOPATHY. *Journal of Jiangsu Clinical Medicine* 2001;5(5):378-380. doi:10.3969/j.issn.1672-2353.2001.05.006

52. Liu Y, Chen Q, Chen W. Clinical significance of serum TNF-α and IL-6 determination in children with viral myocarditis. *Tianjin Med J*. 2001;29(9):552-553. doi:10.3969/j.issn.0253-9896.2001.09.017

53. Lin L, Wang X, Liang Y, Zhang L, Yu Y. Detection of cellular immune function in patients with dilated cardiomyopathy and viral myocarditis. *Journal of Cardiovascular and Pulmonary Diseases*. 2001;20(4):231-232. doi:10.3969/j.issn.1007-5062.2001.04.013

54. Cao J, Pang J, Liu S. Clinical significance of detection of nitrogen monoxide and tumor

necrosis factor contents in patients with viral myocarditis. *Journal of Clinical Cardiology*. 2001;014(2):16-17.

55. Ding Y, Zhang F, Deng S. Detection of serum cytokines in children with viral myocarditis in acute stage and its significance. *Journal of Clinical Pediatrics*. 2000;18(4):211-213. doi:10.3969/j.issn.1000-3606.2000.04.010

56. Sun J, Han Y, Li S, Fu W, Lu J. Detection and clinical significance of serum nitric oxide and tumor necrosis factor in children with viral myocarditis. *Journal of Clinical Pediatrics*. 1998;(06):400-401.

57. Liu X, Gao H, Li Y, Bai C, Li R. VARIANT OF T LYMPHOCYTE SUBSETS AND SERUM TUMOR NECROSIS FACTOR OF CHILDREN WITH VIRAL MYOCARDITIS. *Cellular & molecular immunology*. 1998;(5):73-75.

58. Li W, Yin Y, Wu y. Changes of IL-1 and TNF in acute viral myocarditis and primary dilated cardiomyopathy. *Journal of Norman Bethune University of MedicalScience*. 1998;(5):495-496.

59. Xiang R, Li R, Chen X, et al. Observation on the changes of serum soluble interleukin-2 receptor and tumor necrosis factor-α activity in viral myocarditis. *Journal of Clinical Pediatrics*. 1997;(5):322-323.

60. Ma H, Mai G, Jiang Z. Correlation between serum TNF level and myocardial enzyme in children with viral myocarditis. *Journal of Changzhi Medical College*. 1996;(4):321-322.

61. Xu L, Chen X, Hua X, Jiang M, Lin Z. Changes of plasma tumor necrosis factor activity in children with viral myocarditis. *Journal of Fujian Medical University*. 1994;(4):395-396.
